# Genome-Wide Identification of *Membrane-Bound Fatty Acid Desaturase* Genes in Three Peanut Species and Their Expression in *Arachis hypogaea* during Drought Stress

**DOI:** 10.3390/genes13101718

**Published:** 2022-09-25

**Authors:** Wenyu Gai, Hua Sun, Ya Hu, Chunying Liu, Yuxi Zhang, Shupeng Gai, Yanchao Yuan

**Affiliations:** Key Lab of Plant Biotechnology in Universities of Shandong Province, College of Life Sciences, Qingdao Agricultural University, Qingdao 266000, China

**Keywords:** *FAD*, *Arachis hypogaea*, unsaturated fatty acid, synteny relationship, drought

## Abstract

As a crop irrigated primarily by rain, the quality and yield of peanuts are significantly limited by drought. To date, many studies have indicated that fatty acid desaturase (*FAD*) genes enhance plant tolerance to drought stresses. In this study, 16, 15, and 31 *FADs* were identified in *Arachis duranensis*, *Arachis ipaensis*, and *Arachis hypogaea*, respectively. All the *FADs* were divided into four subfamilies, which had relatively conserved gene structures, motifs, and domains. The synteny relationships and chromosomal position analysis showed that the *FADs* in subgenome pairs, *A. duranensis*-*A. hypogaea* (AA) and *A. ipaensis*-*A. hypogaea* (BB), were homologous, and their physical locations were consistent. The Ka/Ks results indicated that nine *FAD* genes underwent a purifying selection, and *Ah|FAD3.2* experienced positive selection during tetraploid peanut speciation. Various cis-acting elements related to hormone signaling and stress responsiveness in promoters and the predicted miRNA targeting *Ah|FADs* suggested that these genes play crucial roles in drought tolerance. The expression profiles of *Ah|FADs* in 22 tissues and drought-tolerant and -sensitive cultivars under drought stress suggested that 4 and 6 *FADs* were putative genes related to oil accumulation and drought, respectively. These findings will help provide insight into the potential functional roles of the *FAD* genes, which may aid in dealing with plant drought stress.

## 1. Introduction

Peanut (*Arachis hypogaea* L.), one of the essential plant oil and protein crops globally, is mainly cultivated in tropical and subtropical regions and is largely irrigated by rain [1]. As a dominant commercial agricultural crop, a high yield of peanuts greatly benefits farmers as well as food and feed companies. Nevertheless, as the peanut is mainly irrigated by rain, peanut quality and yield are adversely affected by drought stresses, in turn affecting its physiology, biochemistry, and molecular biology and limiting its full genetic potential [2,3]. Droughts are responsible for low peanut production and result in a loss of approximately 6 million tons worldwide annually [4]. Moreover, droughts are occurring at a higher frequency, for a longer duration, and across a broader range, and are projected to be more severe in the next 30–90 years [5,6]. Therefore, identifying drought-related genes and molecular mechanisms is of great significance for selecting and breeding new drought-resistant peanut varieties.

A previous study has shown that an increase in the unsaturated fatty acid content, containing one or more C=C double bonds, compared with the saturated fatty acid content, enhances the tolerance of plants to environmental stresses, such as drought, salt, cold, and heat [7]. Furthermore, unsaturated fatty acid production is catalyzed by fatty acid desaturase (FAD), which significantly contributes to fatty acid metabolism and the maintenance of plant cell membranes [8,9,10]. Moreover, the unsaturated fatty acid content is an important aspect that affects the nutritional quality and yield of peanuts.

The *FAD* genes were identified in eukaryotes to contain four large branches with distinct functions, namely first desaturases, omega desaturases, front-end desaturases, and sphingolipid desaturases [11,12]. The first desaturases, encoded by Arabidopsis desaturase (ADS) genes, desaturate the saturated acyl chain and form the first double bond [12,13]. Omega desaturases introduce a double bond to the Δ12 or Δ15 position between an existing double bond and the acyl end [12,14]. Front-end desaturases, or the cis or trans Δ8 desaturases, are encoded by sphingoid long-chain bases at Δ8 desaturases (SLDs) [12,15]. Sphingolipid desaturases, which desaturate at the Δ4 position and hydroxylate at the C4 position, are also known as dihydroceramide desaturases (DSDs) [16].

To date, many membrane-bound *FAD* genes in various plant species have been identified and demonstrated to respond to adverse environmental conditions. In *Arabidopsis*, *ADS* maintains the polyunsaturated fatty acids in chloroplast lipids, helps form chloroplast membranes, and aids survival under chilling stress [17,18]. Previous studies have indicated that omega desaturases, including ω-3 and ω-6, enhance the stress tolerance of different plants under various conditions, such as salt, cold, and drought [19,20,21,22,23]. For example, ω-3 desaturases, comprising *FAD3*, *FAD7*, and *FAD8*, have enhanced chilling tolerance in *Arabidopsis* [19,24]. Additionally, the overexpression of *LeFAD3* in tomatoes could enhance its resistance to salinity stress [21]. Similarly, the overexpression of *LeFAD3* or *LeFAD7* could improve the cold tolerance of tomatoes [22,25]. In tobacco, the overexpression of either *FAD3* or *FAD8* increases the drought tolerance of transgenic lines [23]. Furthermore, the expression of ω-6 desaturase genes *FAD2* and *FAD6* in *Arabidopsis* seedlings is upregulated under salt stress [26,27]. In peanuts, four *AhSLDs* are induced and upregulated under different degrees of cold and salt stress, and *AhDSD* is highly upregulated under salt stress [28].

In this study, all the *FAD* genes in *Arachis duranensis*, *Arachis ipaensis*, and *A. hypogaea* were identified and characterized. Moreover, these *FAD* genes were analyzed in terms of phylogeny, chromosome localization, gene structures, and gene duplication. The promoter regulatory elements of *Ah|FADs* were analyzed, and the miRNAs targeting these genes were predicted. The expression profiles of the *FAD* genes in 22 tissues were determined in *A. duranensis.* Furthermore, their expression profiles were compared between NH5 (drought-tolerant) and FH18 (drought-sensitive) varieties under drought stress. These results may greatly help understand the structure, phylogeny, and function of the *FAD* gene family in three peanut species and provide a valuable resource for breeding new drought-tolerant or highly nutritional varieties of peanuts.

## 2. Materials and Methods

### 2.1. Sequence Retrieval

The genome and annotation gff3 files of *A. duranensis* (*Ad*, GCF_000817695.2), *A. ipaensis* (*Ai*, GCF_000816755.2), and *A. hypogaea* (*Ah*, GCA_003086295.2) were downloaded from the NCBI database (https://www.ncbi.nlm.nih.gov/assembly/ (accessed on 22 July 2020)). The published FAD protein sequences of *Arabidopsis* *thaliana* [11] and *Oryza sativa* [29] were obtained from the TAIR (release 10, The Arabidopsis Information Resource (TAIR). Available online: http://www.arabidopsis.org/index.jsp (accessed on 22 July 2020)) and RGAP (release 7, Rice Genome Annotation Project (RGAP). Available online: http://rice.plantbiology.msu.edu/index.html (accessed on 22 July 2020)) databases, respectively. A BLASTP search was run to identify the candidate FADs of the three peanut species with an e-value < 1 × 10^−5^ using the queries of *A. thaliana* and *O. sativa* FAD protein sequences.

### 2.2. Genome-Wide Identification and Phylogenetic Construction of FAD Genes

All candidate FAD protein sequences were further identified using the CDD (NCBI Conserved Domain Database. Available online: https://www.ncbi.nlm.nih.gov/cdd (accessed on 27 July 2020)) with an automatic model and default parameters (threshold = 0.01, maximum hits = 500) and confirmed in InterPro (InterPro. Available online: http://www.ebi.ac.uk/interpro (accessed on 27 July 2020)). The conserved domains of confirmed FAD protein sequences were filtered from the CDD results.

For the phylogenetic tree construction, the FAD protein sequences were first aligned with ClustalW using default parameters. The maximum likelihood tree was built with Mega X [30] using the Poisson model and 1000 replicates bootstrap. Lastly, the tree was colored using ITOL [31].

### 2.3. Characterization of FAD Genes

The physical location and strain of all the *FAD* genes were analyzed using TBtools [32] with the genome annotation gff3 files. The number of amino acids (NAA), molecular weight (Mw), charge, isoelectric point (pI), and the grand average of hydropathy (GRAVY) of protein sequences were analyzed in ProtParam [33]. Their subcellular localizations were obtained from the webserver CELLO v2.5 [34].

### 2.4. Gene Structure and Conserved Motif Analysis

Based on the genome annotation gff3 files, the gene structures of all the *FAD* genes were detected and pictured with TBtools [32]. The conserved motifs of the *FADs* were detected in the webserver MEME v5.1.0 [35] with zoop (zero or one occurrence per sequence) in site distribution, 6 to 50 as the width of motifs, and 35 as the maximum number of motifs. Finally, the visualizations of the phylogenetic tree, conserved motifs, conserved domains, and gene structures were constructed and merged in TBtools [32].

### 2.5. Duplication and Synteny Analysis of FAD Genes

The chromosome localization of all the *FAD* genes was visualized using TBtools based on their location information. For the synteny analysis, the genome sequences of the three peanut species were first compared in pairs using BLAST. Then, synteny was examined, and paralogous genes in the three peanut species were visualized in TBtools. Furthermore, the ratio of the number of nonsynonymous substitutions per nonsynonymous site (Ka) to the number of synonymous substitutions per the synonymous site (Ks) of the homologous genes was calculated using the “Simple Ka/Ks Calculator” in TBtools [32].

### 2.6. Cis-Element Prediction in Promoter Regions and Expression Analysis of Ah|FAD Genes

To predict the stress-related cis-acting regulatory elements in promoter sequences, the 1.5 kb upstream regions (from translation starting sites) of the *FAD* genes were extracted and analyzed in the PlantCARE database [36,37].

The expression profiles of the *FAD* genes in 22 *A. hypogaea* tissues were obtained from the tissue expression atlas in the PeanutBase webserver [38]. Twenty-two *A. hypogaea* tissues were obtained from the seeding leaf 10 d post emergence (leaf 1), the central stem leaf (leaf 2), the lateral (n + 1) leaf (leaf 3), the vegetative shoot tip from the main stem (veg shoot), the reproductive shoot tip from the first lateral (n + 1) (repr shoot), 10 d roots (root), 25 d nodules (nodule), perianth, gynoecium (pistil), androecium (stamen), the aerial gynophore tip (peg tip 1), the subterranean gynophore tip (24 h) (peg tip 2), Pattee 1 pod (fruit Pat. 1), Pattee 1 stalk (peg tip Pat. 1), Pattee 3 pod (fruit Pat. 3), Pattee 5 pericarp (pericarp Pat. 5), Pattee 5 seed (seed Pat. 5), Pattee 6 pericarp (pericarp Pat. 6), Pattee 6 seed (seed Pat. 6), Pattee 7 seed (seed Pat. 7), Pattee 8 seed (seed Pat. 8), and Pattee 10 seed (seed Pat. 10). Furthermore, the expression profiles of the *FADs* in the second compound leaves were compared between NH5 (drought-tolerant) and FH18 (drought-sensitive) varieties, which were drought-treated for 0 h (CK), 4 h (DT1), 8 h (DT2), and 24 h (DT3) [6]. The SRA data were downloaded from the NCBI database (SRA accession: PRJNA657965) and analyzed in TuxNet software with *A. hypogaea* as the reference genome [39].

Furthermore, the expressions of the putative genes related to drought tolerance were tested using qRT-PCR of ‘Huayu22’ (drought-tolerant) and ‘Huayu23’ (drought-sensitive) under drought and water treatments. The drought resistances of ‘Huayu22’ and ‘Huayu23’ were identified and evaluated in previous studies [40,41]. After the total RNA was extracted with an OminiPlant RNA Kit (CWBIO), 1 µg was used to synthesize the template cDNA in a 20 µL reaction volume with a HiFiScript cDNA Synthesis Kit (CWBIO). *AhActin* was used as an internal reference, and all the primers are shown in Table S1. The Applied Biosystems 7500 Real-Time PCR System was employed. Each reaction was performed at least three times, and the relative expressions were analyzed with the ΔΔCt method.

### 2.7. Assays for Water Content and Antioxidative Enzymes Activity

The plants of ‘Huayu22’ (drought-tolerant) and ‘Huayu23’ (drought-sensitive) under drought and water treatments were weighed (fresh weight) or dried for 48 h at 80 °C and then weighed (dry weight). Then, that water content was calculated as follows: Water content = (fresh weight − dry weight)/dry weight × 100%.

The heart leaves of ‘Huayu22’ and ‘Huayu23’ under drought and water treatments were sampled and homogenized in liquid nitrogen. The activities of catalase (CAT), superoxide dismutase (SOD), and peroxidase (POD) were measured using a catalase assay kit, a superoxide dismutase assay kit, and a peroxidase assay kit (Solarbio, Beijing, China), respectively. The experiments were carried out according to the instructions.

### 2.8. Prediction of miRNA Targeting Ah|FAD Genes

The miRNAs targeting *Ah|FAD* genes were predicted by querying their whole coding sequences against the 2017 updated miRbase database in psRNATarget [42]. The default parameters were used with the maximum expectation modified to 3.0. Further interaction networks between miRNAs and *Ah|FADs* were illustrated using the Cytoscape version 3.7.0 (USA) software [43].

## 3. Results

### 3.1. Identification and Phylogenetic Analysis of FAD Genes in Three Peanut Species

To detect the FADs in the three peanut species, the AtFAD and OsFAD protein sequences were used as queries in the BLASTP search. The protein sequences of nonredundant candidate peanut FADs were then submitted to CDD and InterPro to confirm the real *FAD* genes with conserved domains. In total, 16, 15, and 31 *FADs* were identified in *A. duranensis* (AA), *A. ipaensis* (BB), and *A. hypogaea* (AABB), respectively (Table S2).

**Figure 1:**
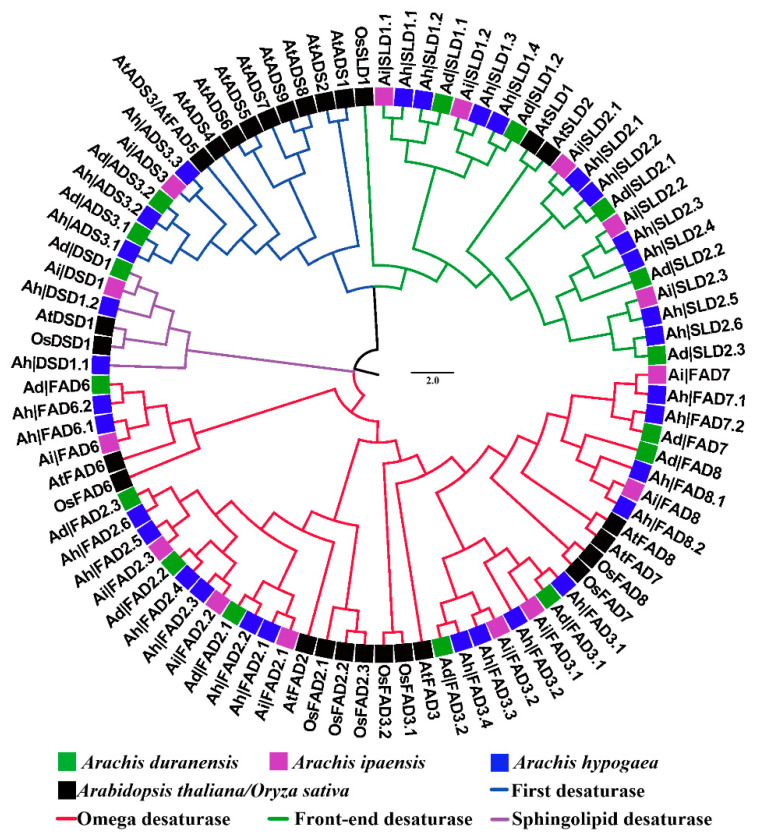
Phylogenetic tree of *FAD* genes from three peanuts species, *Arabidopsis thaliana*, and *Oryza sativa*.

To analyze gene subfamilies and phylogeny, a phylogenetic tree of all the FAD protein sequences in the three peanut species, *A. thaliana*, and *O. sativa* was constructed (Figure 1). As shown in the evolutionary tree, all the FAD proteins of each peanut species were divided into four subfamilies, namely the first desaturase (ADS), the omega desaturase (FAD2, 3, 6, 7, and 8), the frond-end desaturase (SLD), and the sphingolipid desaturase (DSD), and renamed according to their homology with AtFADs/OsFADs (Table S2). At the end of the phylogenetic tree clades, four genes from the three peanut species were clustered together in most branches, whereas six genes, i.e., *Ah|ADS3.1*, *Ad|ADS3.1*, *Ah|ADS3.2*, *Ad|ADS3.2*, *Ah|ADS3.3*, and *Ai|ADS3*, were gathered in one cluster. With higher sequence similarity and closer genetic relationship, the genes in the same cluster were homologous to each other (Figure 1).

### 3.2. Characterization Analysis of FAD Genes in Three Peanut Species

To further understand the structure and function of *FADs*, chromosomal location, NAA, Mw, charge, pI, and GRAVY were determined and are shown in Table S2. The NAA, Mw, and pI were approximately equal in each classification of ADSs, DSDs, FAD6, FAD7, FAD8, and SLD, with the average values of 389.33, 334.00, 442.00, 455.00, 454.25, and 449.25 in NAA, respectively; 44.91 kDa, 38.88 kDa, 51.62 kDa, 51.62 kDa, 52.96 kDa, and 51.74 kDa in Mw, respectively; and 9.19, 7.87, 9.09, 8.35, 7.69, and 8.69 in pI, respectively. Exceptionally, the NAA, Mw, and pI of SLD1.2 were significantly different from those of other SLD proteins. Additionally, the NAA, Mw, and pI of FAD2 proteins ranged from 349 to 421, from 39.74 kDa to 51.64 kDa, and from 8.80 to 9.09, respectively; and those of FAD3 varied from 375 to 442, from 43.74 kDa to 52.97 kDa, and from 7.52 to 8.95, respectively. Furthermore, the GRAVY of all FAD proteins was < zero, except for that of FAD2.2 and all eight SLD1s, indicating that FAD2.2 and SLD1s were hydrophobic proteins. The subcellular localizations were also predicted using the webserver CELLO v2.5. The ADSs, DSDs, and SLDs were located in the plasma membrane, whereas the FAD*2*~3s and FAD6~8s were located in the endoplasmic reticulum (ER) and chloroplast, respectively. Overall, these results indicated that the *FAD* genes of the same category were similar.

### 3.3. Analysis of Gene Structure of FAD Genes and Domains and Motifs of Their Encoding Proteins

**Figure 2:**
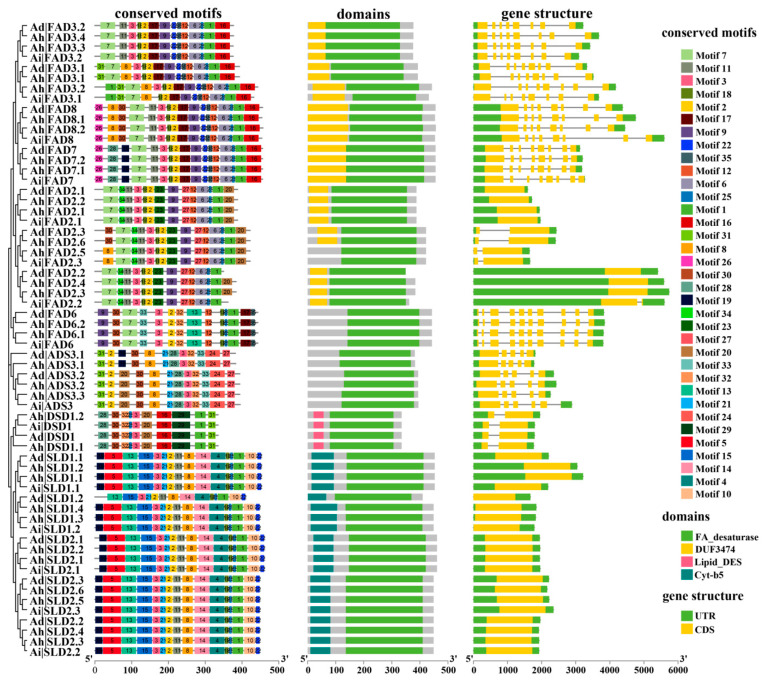
Gene structure of *FAD* genes and conserved motifs and domains of their encoding proteins in three peanut species.

A structural examination of *FADs* (Figure 2) showed that all the *SLDs* and half of the *FAD2s* had only one exon, whereas the others had multiple exons. Moreover, the *ADSs* had five exons, whereas the ω-3 desaturase genes (*FAD3*, *FAD7*, and *FAD8*) and the *FAD6s* had eight and ten exons, respectively. These results showed that the gene structures of the same subfamily were similar, such as in the number and length of their introns and exons.

The conserved domains of FAD proteins were detected with CDD during the identification of the *FAD* genes as described above. The conserved motifs were dissected in the MEME web with their full-length protein sequences. All FAD proteins had the FA desaturase domain, all DSDs and SLDs had both Cyt-b5 and lipid desaturase domains, respectively, and the majority of FADs had the DUF3474 domain (Figure 2). Moreover, FADs contained a total of 35 conserved motifs, which consisted of 8–50 amino acids (Figure 2; Table S3). In detail, the number of conserved motifs was divergent in the *FAD* genes, ranging from 10 to 17. The number and sets of conserved motifs were similar in the same subgroup of FAD proteins. For example, the ω-3 and ω-6 desaturase proteins had motif set 1 and motif set 2, respectively, and motif set 3 was shared in the ω desaturases (Table 1). However, the *FAD6* proteins had their specific motifs, which were different from those of *FAD2*. Additionally, ADSs, DSDs, and SLDs had their conserved motif sets.

### 3.4. Chromosome Localization and Synteny Analysis of FAD Genes

**Figure 3:**
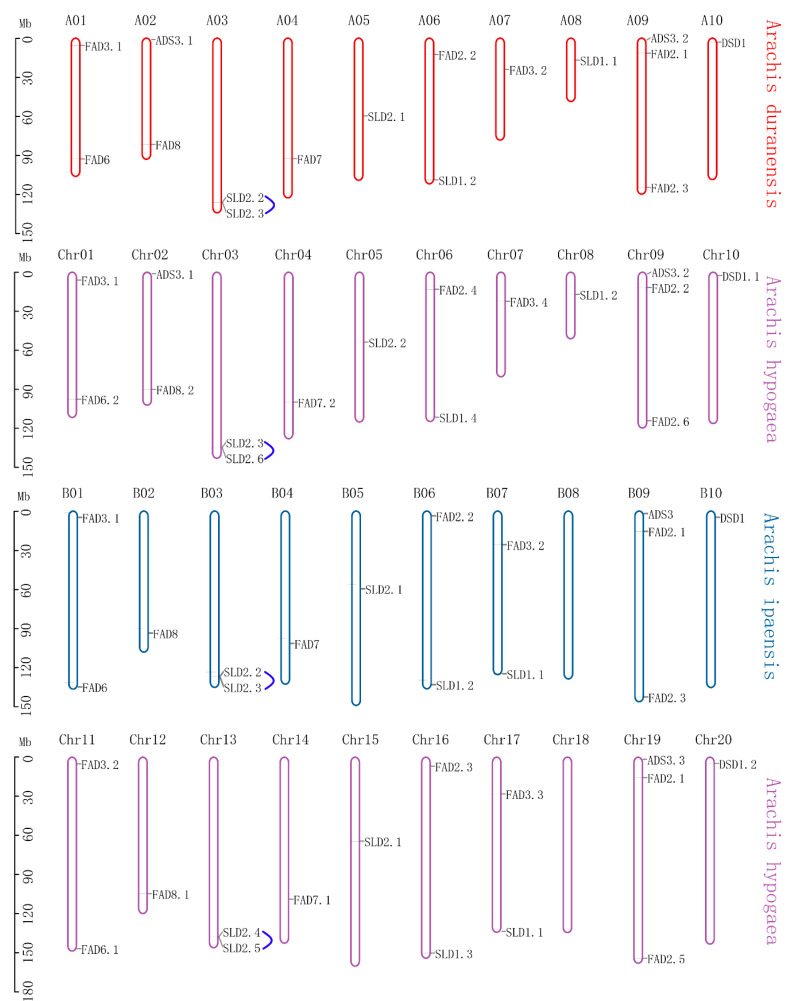
Chromosome distribution of FAD genes in three peanut species.

The chromosomal location of the gene provides an essential reference for dissecting the evolution and function of the gene family. In this study, the physical location of the *FAD* genes in the three peanut species was visualized (Figure 3). All chromosomes contained the *FAD* genes except B08 in *Ai* and Chr18 (B08) in *Ah*. The numbers of *FADs* in subgenomes were identical in *Ad* (AA), *Ai* (BB), and *Ah* (AABB), and their physical locations in subgenomes were consistent.

**Figure 4:**
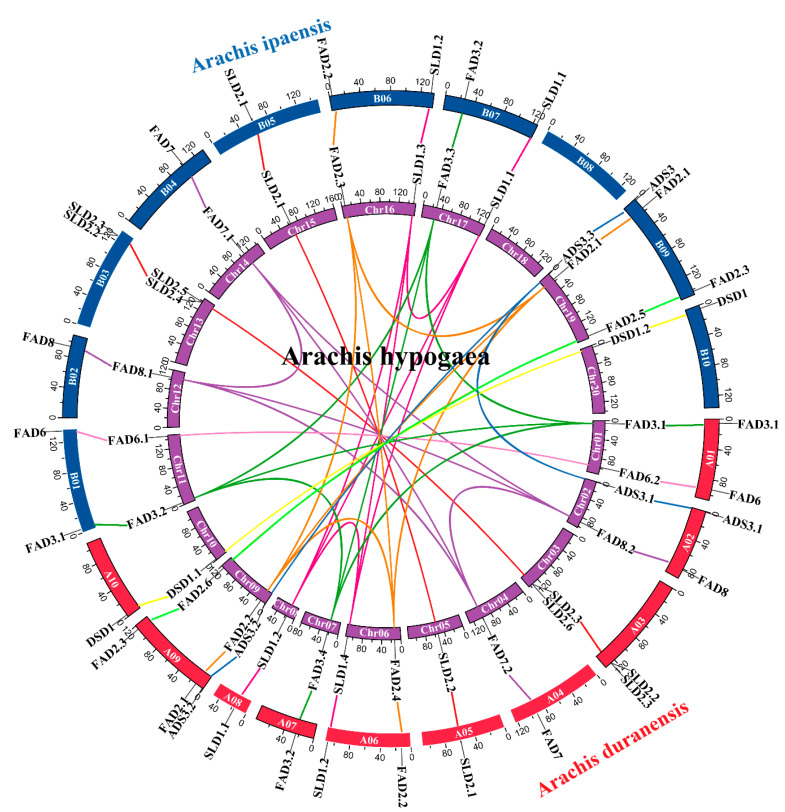
Synteny of FAD genes among *Arachis duranensis*, *Arachis ipaensis*, and *Arachis hypogaea*.

The whole genomes of the three peanut species were aligned and analyzed to dissect the synteny relationships, and the linked gene pairs were identified (Figure 4; Table S4). All 31 *Ah|FADs* had collinear genes in *Ai* and *Ad*, except *Ah|SLD2.5* and *Ah|SLD2.5*; consequently, all the *FADs* in *Ai* and *Ad* had collinear genes in *Ah*, except *Ad|SLD2.3* and *Ai|SLD2.3.* During the evolution of plants, duplicate mechanisms indispensably contributed to the expansion of gene families [44,45]. “Tandem replication event” and “singleton” indicate the appearance of two or more similar genes in the same 200 kb chromosomal region and a single-copy gene, respectively. “Dispersed” indicates that the gene may arise from transposition, such as “replicative transposition,” “nonreplicative transposition,” or “conservative transposition,” whereas “whole-genome duplication (WGD)” or “segmental duplication” show that the gene might arise from whole-genome or segmental duplication [46]. *Ad|DSD1*, *Ai|DSD1*, and *Ai|ADS* belonged to the “singleton” genes in their genomes, whereas the gene pairs of *Ad|SLD2.2*-*Ad|SLD2.3*, *Ai|SLD2.2*-*Ai|SLD2.3*, *Ah|SLD2.4*-*Ah|SLD2.5*, and *Ah|SLD2.3*-*Ah|SLD2.6* were tandem replication events (Table S5). In the diploids, seven *Ad* and three *Ai FADs* were regarded as dispersed events, whereas the others were WGD or segmental events. All the *Ah|FADs* were considered as WGD or segmental duplications.

To further understand gene selection in the *A. hypogaea* speciation, the Ka/Ks ratios of *FAD* pairs between diploid and tetraploid peanuts were calculated (Table S6). *Ah|FAD3.2* might be caused by positive selection from *Ai|FAD3.1*, whereas *Ah|ADS3.1*, *Ah|ADS3.2*, *Ah|DSD1.1*, *Ah|FAD2.4*, *Ah|FAD2.5*, *Ah|FAD2.6*, *Ah|FAD8.2*, and *Ah|SLD2.3* were caused by purifying selection during the formation of *Ah* from diploid genomes. The other genes arose from neutral evolution.

### 3.5. Stress-Related Cis-Elements in the Promoters of Ah|FAD Genes

**Figure 5:**
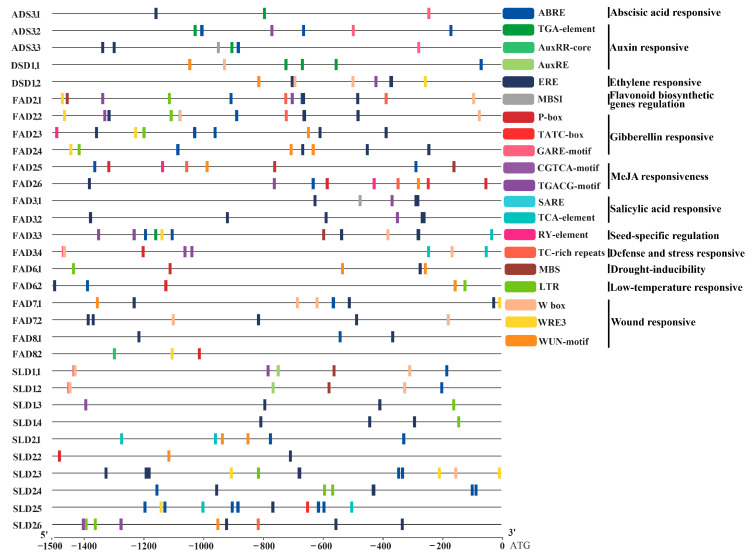
Predicted hormone responsiveness or stress-related cis-elements in *Ah|FAD* promoters (1500 bp upstream region).

To further explore the regulation of *Ah|FAD* gene expression under stress conditions, the cis-elements of the *Ah|FAD* promoter regions were analyzed. The elements related to hormones or stress are illustrated in Figure 5. The potential cis-elements of *Ah|FAD* genes were involved in various responses to hormones (abscisic acid (ABA), auxin, ethylene, flavonoid, gibberellin (GA), jasmonic acid/methyl jasmonate (MeJA), and salicylic acid (SA)) and stress (defense and stress, drought, low-temperature, and wound). In total, 31 *Ah|FAD* promoters containing cis-elements were related to stress (25) and hormones (31). In detail, 5 defense and stress-responsive elements (TC-rich repeats), 5 drought-inducibility elements (MBS), 11 low-temperature responsive elements (LTR), and 31 wounding responsive elements (11 WUN-motif, 12 W box, and 10 WRE3) were found in *Ah|FAD* promoters (Figure 5), indicating the roles of related genes in regulatory networks under various stresses. Additionally, 19, 8, 23, 2, 13, 12, and 5 promoters containing elements responded to ABA, auxin, ethylene, flavonoid, GA, MeJA, and SA, respectively, suggesting that *Ah|FADs* play vital roles in the plant hormone signaling network leading to stress responsiveness. Furthermore, a seed-specific regulation element (RY-element) was found in *Ah|ADS3.1* and *Ah|FAD3.1* promoters.

### 3.6. Expression Profile Analysis of Ah|FAD Genes

**Figure 6:**
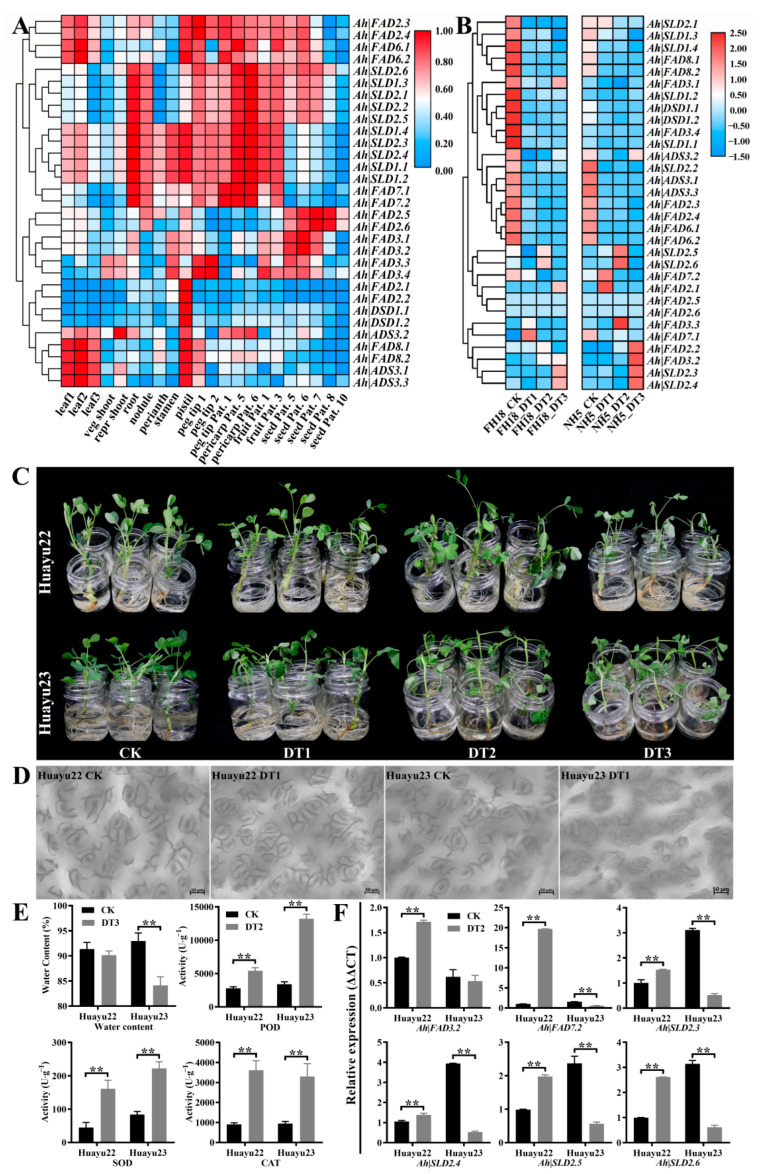
The expression analysis of *Arachis hypogaea FAD* genes: (**A**) transcriptome expression of *Arachis hypogaea FAD* genes in 22 tissues. Seeding leaf 10 d post-emergence: leaf 1; main stem leaf: leaf 2; lateral (n + 1) leaf: leaf 3; vegetative shoot tip from main stem: veg shoot; reproductive shoot tip from first lateral (n + 1): repr shoot; 10 d roots: root; 25 d nodules: nodule; perianth, gynoecium: pistil; androecium: stamen; aerial gynophore tip: peg tip 1; subterranean gynophore tip (24 h): peg tip 2; Pattee 1 pod: fruit Pat. 1; Pattee 1 stalk: peg tip Pat. 1; Pattee 3 pod: fruit Pat. 3; Pattee 5 pericarp: pericarp Pat. 5; Pattee 5 seed: seed Pat. 5; Pattee 6 pericarp: pericarp Pat. 6; Pattee 6 seed: seed Pat. 6; Pattee 7 seed: seed Pat. 7; Pattee 8 seed: seed Pat. 8; Pattee 10 seed: seed Pat. 10; (**B**) transcriptome expression of *FAD* genes in drought-sensitive (FH18) and -tolerant (NH5) species under drought conditions. CK (0 h), DT1 (4 h), DT2 (8 h), and DT3 (12 h) indicate treatment with 20% PEG6000 for 0 h, 4 h, 8 h, and 24 h, respectively; (**C**) phenotypic analysis of Huayu23 (drought-sensitive) and Huayu22 (drought-tolerant) seedlings at CK (0 h), DT1 (4 h), DT2 (8 h), and DT3 (12 h) conditions; (**D**) stomatal observations of Huayu23 and Huayu22 leaves at CK and DT1 conditions on a Zeiss fluorescence positive microscope (Axio Scope A1); (**E**) the water-holding capacity and the antioxidative enzyme (SOD, POD, and CAT) activity in Huayu23 and Huayu22 with or without drought stress; ** indicates a significant difference at *p* < 0.01; (**F**) qRT-PCR results of six *FAD* genes in drought-sensitive (Huayu23) and -tolerant (Huayu22) species under drought or normal water conditions; ** indicates a significant difference at *p* < 0.01. Furthermore, the expression profiles of *FADs* were compared between NH5 (drought-tolerant) and FH18 (drought-sensitive) varieties after drought treatment for 0 h (CK), 4 h (DT1), 8 h (DT2), and 24 h (DT3) (Figure 6B). *Ah|FAD7.2*, *Ah|FAD2.1*, *Ah|FAD2.2*, *Ah|FAD3.2*, *Ah|FAD3.3*, *Ah|SLD2.3*, *Ah|SLD2.4*, *Ah|SLD2.5*, and *Ah|SLD2.6* were highly expressed in drought-treated NH5 compared with NH5 without drought treatment and FH18 with or without drought treatment. *Ah|FAD2.5* and *Ah|FAD2.6* showed low expression levels in all treatments. All other genes were mainly expressed in untreated FH18 and untreated NH15.

The gene expressions of all *Ah|FADs* in the 22 tissues were analyzed and are shown in Figure 6A. The results showed that the genes were preferentially expressed in the different tissues and were gathered into seven expression patterns. In detail, *Ah|FAD2.3*, *Ah|FAD2.4*, *Ah|FAD6.1*, and *Ah|FAD6.2* were expressed at higher levels in the leaves, pistil, peg tips, pericarp Pat., fruit Pat., and seed Pat. 5–6, whereas *Ah|SLD2.6*, *Ah|SLD1.3*, *Ah|SLD2.1*, *Ah|SLD2.2*, and *Ah|SLD2.5* were preferentially expressed in the root, nodule, peg tips, pericarp Pat., fruit Pat., and seed Pat. 5–8. The expression patterns of the other five *Ah|SLDs*, *Ah|FAD7.1*, and *Ah|FAD7.2* were similar to those of the previous five *SLDs*. Additionally, the expression levels of the five *Ah|SLDs* were higher in leaf 1–2 and lower in seed Pat., and *Ah|FAD7.1* and *Ah|FAD7.2* were lowly expressed in seed Pat. 5–10. *Ah|FAD2.5*, *Ah|FAD2.6*, *Ah|FAD3.1*, and *Ah|FAD3.2* were preferentially expressed in seed Pat., whereas *Ah|FAD3.3* and *Ah|FAD3.4* were highly expressed not only in fruit Pat. and seed Pat. but also in stamen and peg tips. Moreover, *Ah|FAD2.1*, *Ah|FAD2.2*, *Ah|DSD1.1*, and *Ah|DSD1.2* were only expressed in the pistil, whereas *Ah|ADS3.1*, *Ah|ADS3.2*, *Ah|ADS3.3*, *Ah|FAD8.1*, and *Ah|FAD8.2* were expressed in both leaves and pistil.

**Figure 7:**
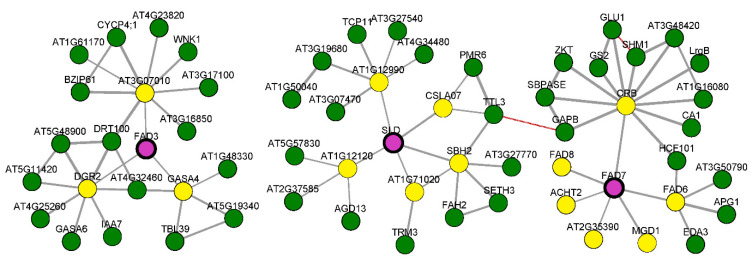
Co-expression of homologous genes of putative drought-related *Ah|FADs* in *Arabidopsis thaliana*.

Based on the comprehensive analysis of the two expression profiles, *Ah|FAD3.2*, *Ah|FAD7.2*, *Ah|SLD2.3*, *Ah|SLD2.4*, *Ah|SLD2.5*, and *Ah|SLD2.6* were regarded as putative drought-related genes. For further validation, ‘Huayu22’ (drought-tolerant) and ‘Huayu23’ (drought-sensitive) were selected for drought and normal water treatments (Figure 6C–F). After drought treatment, ‘Huayu23’ leaves wilted earlier and more severely than those of ‘Huayu22’ (Figure 6C). In addition, the closure patterns of stomata, which control carbon and water exchange between the leaf surface and the atmosphere, were observed, and the drought conditions induced a quicker stomatal closure in ‘Huayu23’ leaves than in ‘Huayu22’ leaves (Figure 6D). Furthermore, the water content of ‘Huayu23’ significantly decreased after drought treatment, while that of ‘Huayu22’ did not (Figure 6E). All these observations indicated that ‘Huayu22’ could preserve higher leaf/plant water contents than ‘Huayu23’ under drought conditions. Then, the activities of SOD, POD, and CAT were determined to present the differences between drought and normal treatments. The results showed that the antioxidative enzyme activities were significantly induced by drought in both ‘Huayu22’ and ‘Huayu23’, and SOD and POD were higher in ‘Huayu23’, suggesting that the ‘Huayu23’ (drought-sensitive) were suffering more damage from drought stress than ‘Huayu22’ (Figure 6E). Meanwhile, qRT-PCR was employed to detect the expressions of the six putative genes in the two varieties under drought and standard water treatments (Figure 6F). As shown, under drought stress, all six genes were upregulated in drought-tolerant ‘Huayu22’ but downregulated in drought-sensitive ‘Huayu23’, indicating that they could respond to drought. Furthermore, the homologous genes of these six *Ah|FADs* in *A. thaliana* were employed to detect the co-expressed genes on the ATTED-II webserver [47]. This investigation revealed that these six *Ah|FADs* were tightly co-regulated with their direct targets *GASA4*, *AT1G71020*, *SBH2*, *ACHT2*, *FAD6*, *FAD8*, and *CRB* across the public experimental microarray datasets (Figure 7). *GASA4* and *AT1G71020* were the genes involved in gibberellic-acid- and jasmonic-acid-mediated signaling pathways, respectively. *SBH2*, *ACHT2*, *FAD6*, and *FAD8* were involved in the oxidation–reduction process, and *FAD8* also responds to temperature stimulus. In addition, the *CRB* responds to various stresses, such as water deprivation, wounding, cold, and bacteria. These results indicated that the six candidate *FAD* genes were associated with drought.

### 3.7. The miRNA Targeting Ah|FAD Genes

**Figure 8:**
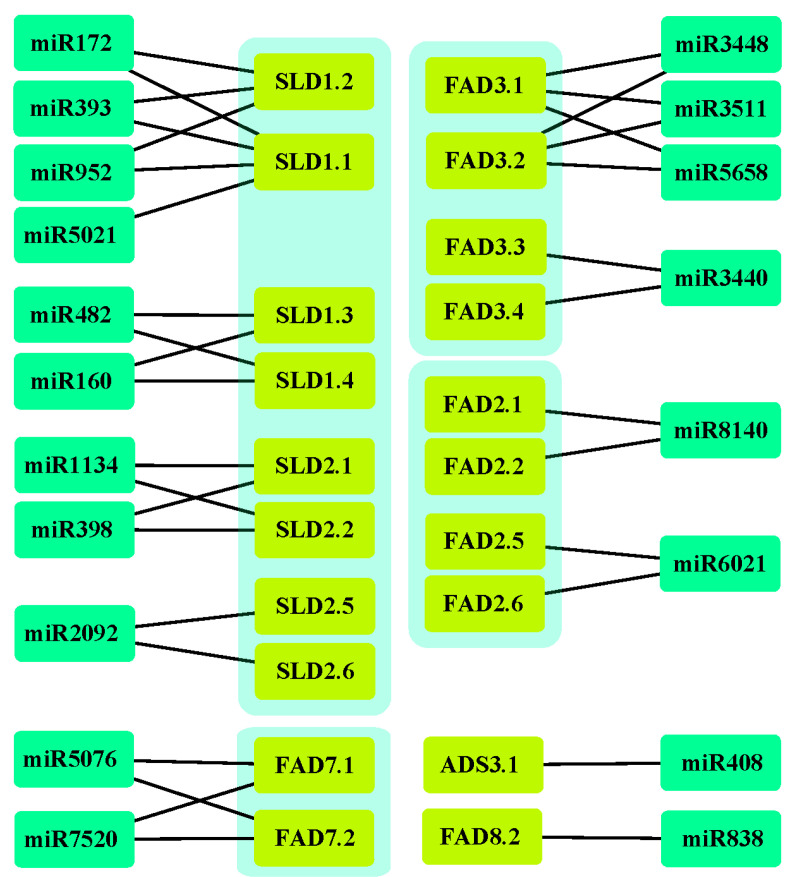
Interaction network of miRNAs and their target *Ah|FADs*.

To dissect the regulation of *Ah|FAD* expression, the putative miRNAs targeting the *FAD* genes were predicted using the psRNATarget server with all the published miRNAs in various species. In total, 19 miRNAs targeting 20 *Ah|FADs* with expectations lower than 3.0 were identified (Figure 8). The details are shown in Table S7. Our results revealed that only *Ah|DSDs* were not targeted by miRNAs, and the most targeted *Ah|FAD* genes were the FAD type, consisting of four *FAD3s*, four *FAD2s*, two *FAD7s*, and one *FAD8*, followed by eight *Ah|SLDs* (Figure 8). Moreover, only miR3511 belongs to *A. hypogaea.*

## 4. Discussion

Drought significantly and negatively affects the growth and yield of peanuts [4,6]. Previous studies have shown that FADs, which catalyze the formation of unsaturated fatty acids, respond to various stress, such as drought, salt, cold, and heat [7,28]. Therefore, the excavation and identification of the *FAD* genes are important and can be applied to improve crop yield and quality. At present, the whole-genome identification of the *FAD* genes has been performed in many plants, such as *Gossypium hirsutum* [11], *O. sativa* L. [29], *Medicago truncatula* [48], and *Brassica napus* [49]. Multi-generation sequencing and genome assembly make it possible for the whole-genome analysis of the FAD gene family in peanuts. *A. hypogaea*, the allotetraploid species, is a cultivated peanut widely planted in Asia, America, and Africa for vegetable oil and protein. Moreover, the origin of *A. hypogaea* (AABB) was proposed to be the result of an initial hybridization of *A. duranensis* (AA) and *A. ipaensis* (BB) [50,51]. Therefore, to explore the origins and consequences of the *Ah|FAD* gene family, we compared it to the *Ad|FAD* and *Ai|FAD* gene families. In addition, the cis-regulatory elements in the promoters, the expression levels under drought, the miRNA–mRNA network, and the co-expressed genes of *Ah|FADs* were analyzed to dissect the drought-related *FADs* and improve the drought tolerance of the cultivated peanut.

In this study, 16, 15, and 31 *FADs* were identified in *A. duranensis*, *A. ipaensis*, and *A. hypogaea*, respectively, and named according to their homology with *AtFADs*/*OsFADs* (Table S2, Figure 1). In a previous study, 36 *FADs* were identified in *A. hypogaea*, which contained five pairs of alternatively spliced transcripts [52], and the number of *FAD* genes identified was consistent with this study. This study also found that chromosome 08 of *A. ipaensis* was longer than that of *A. duranensis* and lacked one *FAD* gene (Figure 3), which might be due to the greater frequency of local duplications and higher transposon content in *A. ipaensis* (BB) than *A. duranensis* (AA) in the process of evolution [51]. Moreover, the total number of *FAD* genes in *A. hypogaea* was equal to their sum in *A. duranensis* and *A. ipaensis*, supporting that the allotetraploid peanut originated from the two diploid ancestors [51]. Evolution analysis showed that the *FAD* genes of the three peanut species were all divided into four subfamilies (Figure 1). At the end of their phylogenetic trees, two *A. duranensis* genes, one *A. ipaensis* gene, and one *A. hypogaea* gene were clustered together and formed sister pairs, which were homologous to each other. *Ah|ADS3.1*, *Ad|ADS3.1*, *Ah|ADS3.2*, *Ad|ADS3.2*, *Ah|ADS3.3*, and *Ai|ADS3* were also clustered. In detail, the genes in each of the clusters consisted of one Ad (A subgenome) gene, one *Ai* (B subgenome) gene, one *Ah* (A subgenome) gene, and one *Ah* (B subgenome) gene. This result was also consistent with *Ah* (tetraploids) from *Ad* and *Ai* (two diploids) [51].

The chemical properties (NAA, Mw, and pI) of the proteins encoded by *ADSs*, *DSDs*, *FAD6s*, *FAD7s*, *FAD8s*, and *SLDs* were similar in the same gene category, with the exception of a few genes (Table S2). Moreover, most FAD proteins were hydrophilic, whereas FAD2.2 and SLD1s were hydrophobic. These properties might lead to their similar and varied functions in the same or different gene categories. Furthermore, the location of the FADs was predicted, and ADSs, DSDs, and SLDs were found to be distributed in the plasma membrane, whereas FAD2/3s and FAD6/7/8s were located in the ER and chloroplast, respectively. In a previous study, experiments showed that four AhFAD3s were concentrated in the ER but were also detected in the cytoplasm and cell membranes [52]. In addition, these inferred results of localization matched with their function and location as provided in previous studies. For ER glycerolipids, the conversions of oleic acid (18:1) to linoleic acid (18:2) and linoleic acid (18:2) to linolenic acid (18:3) are catalyzed by FAD2 and FAD3, respectively. In contrast, the plastid glycerolipids are desaturated by FAD6 (18:1 to 18:2) and FAD7/8 (18:2 to 18:3) [53].

The conserved structural domains and motifs of FAD proteins were also determined and are visualized in Figure 2 and Figure 3. Most genes in the same subfamily have a similar number and type of conserved domains and motifs. The shared motif sets in subfamilies might explain the similar functions of these proteins. In contrast, the specific conservative motif sets among subfamilies might lead to their functional diversity. For example, in the ω-6 desaturase proteins, the specific motifs of FAD6 proteins were compared with those of FAD2. The results suggested that FAD6 converts oleic acid (18:1) to linoleic acid (18:2) in plastid glycerolipids, whereas the conversion of ER glycerolipids is catalyzed by FAD2 [26,27]. These results may provide a reference for studying the functional differentiation between subfamilies; they also support the accuracy of the phylogenetic tree constructed in this study.

Gene replication plays an irreplaceable role in expanding a gene family [46]. In diploid peanuts, the genes had all four duplicate mechanisms, and the orthologous genes showed the same duplication forms. In the tetraploid peanut, only tandem events and WGD or segmental events were found (Table S4). In *G. hirsutum*, the expansion of three *FAD* subfamilies was due to segmental duplication [11], which was consistent with this study. Furthermore, all the *GhFADs* had experienced intense negative/purifying selection pressure, contributing to the maintenance of their function [11]. In the formation of tetraploids from diploids, only *Ah|FAD3.2* experienced positive selection, and the others underwent purifying selection or neutral evolution (Table S6), indicating that *Ah|FAD3.2* developed functional variation to adapt to the environment.

The identification of the cis-regulatory elements in the target gene promoters can provide a better understanding of their transcriptional regulation [54]. The majority of *Ah|FADs* encoded at least one type of cis-element responsible for various stress responses in promoter regions, suggesting their diverse roles in different stress regulatory networks (Figure 5). Hormone signaling plays an essential role in the stress resistance of plants, especially ABA, MeJA, and SA [55]. Therefore, the presence of hormone response elements in the promoter regions of *Ah|FAD* genes suggested that they were involved in stress response (Figure 5).

Although the gene structure, conserved domains, and conserved motifs of *FADs* in the same subfamilies were remarkably similar, their expression patterns were divergent. This might be related to their mechanism of adaptation to different environments. In this study, *FADs* were clustered into several large groups containing different types of *FAD* genes based on their expression patterns in the 22 tissues (Figure 6A). The preferentially expressed *FADs* in fruit Pattee and seed Pattee might be regarded as oil-related *FADs*; therefore, *Ah|FAD2.5*, *Ah|FAD2.6*, *Ah|FAD3.1*, and *Ah|FAD3.2* might play an essential role in oil accumulation in peanut. A previous study showed that the ectopic expression of four *AhFAD3s* in *A. thaliana* increased their seed oil and salinity tolerance [52], and tung oilseed *FAD2* was involved in unsaturated fatty acid accumulation in *Rhodotorula glutinis* and *A. thaliana* [19]. Moreover, all the *Ah|SLDs* and *FAD7s* were preferentially expressed in the root. Thus, the four oil-related genes, *Ah|SLDs*, and *FAD7s* might be regarded as candidate genes in response to rhizosphere stress, such as drought, salinity, and waterlogging. Furthermore, the expressions of *Ah|FAD7.2*, *Ah|FAD2.1*, *Ah|FAD2.2*, *Ah|FAD3.2*, *Ah|FAD3.3*, *Ah|SLD2.3*, *Ah|SLD2.4*, *Ah|SLD2.5*, and *Ah|SLD2.6* were induced by drought stress in NH5 but not in FH18 (Figure 6B), indicating that these genes are related to drought tolerance. Combining the two expression profiles and qRT-PCR results (Figure 6C), *Ah|FAD3.2*, *Ah|FAD7.2*, *Ah|SLD2.3*, *Ah|SLD2.4*, *Ah|SLD2.5*, and *Ah|SLD2.6* were the putative genes that respond to drought stress. These agreed with the results of previous studies, which have shown that omega desaturases, including *FAD2* and *FAD3*, enhance the tolerance of plants to various stresses, such as salt, chilling, and drought [19,20,21,22,23]. Additionally, *Ah|SLDs* are upregulated under both cold and salt stress [28]. Furthermore, these six *Ah|FADs* were tightly co-regulated with their direct targets, *GASA4* (gibberellic acid signaling pathway gene), *AT1G71020* (jasmonic acid signaling pathway gene), *SBH2*, *ACHT2*, *FAD6*, *FAD8* (four oxidation–reduction process genes), and *CRB* (responses to water deprivation, wounding, cold, and bacteria) (Figure 7). These findings supported the hypothesis of the candidate drought-related genes.

Dissecting the miRNA–mRNA interaction network helps further understand the regulation of *Ah|FADs* for peanut cultivar improvement. Various miRNA families are involved in plant development and respond to different stresses [56,57], for example, drought response in peanuts [58]. In this study, 19 miRNA families targeting *Ah|FADs* were predicted, including miR160, miR172, miR393, miR398, miR408, miR482, miR838, miR952, miR1134, miR2092, miR3440, miR3448, miR3511, miR5021, miR5076, miR5658, miR6021, miR7520, and miR8140 (Figure 8). Among them, the knockout of miR398 was found to raise plant stress resistance [59]. Moreover, the following predicted miRNAs were also regulated in response to drought: miR160, miR172, miR393, miR398, miR408, and miR482 [60,61,62].

## 5. Conclusions

Based on bioinformatic tools, a comprehensive genome-wide analysis of the peanut FAD gene family was conducted, and systematic identification and functional annotations were provided. A total of 16 *A. duranensis*, 15 *A. ipaensis*, and 31 *A. hypogaea FADs* were identified and characterized. After phylogenetic analysis, the FAD gene family was divided into four subfamilies. Moreover, detailed information on gene structures, chromosome distribution and synteny, and the possible subcellular localizations of *FADs* was provided. The Ka/Ks results showed that most *FAD* genes went through neutral evolution, whereas nine genes underwent purification selection during evolution, and *Ah|FAD3.2* experienced positive selection. The regulatory cis-elements in promoters and miRNA targeting *Ah|FADs* confirmed their essential roles in drought response processes. In addition, the expression profile of *FAD* genes from 22 tissues under normal conditions and in drought-sensitive and -tolerant species under drought stress suggests that *Ah|FAD2.5*, *Ah|FAD2.6*, *Ah|FAD3.1*, and *Ah|FAD3.2* are related to the accumulation of fatty acids in peanut, and *Ah|FAD3.2*, *Ah|FAD7.2*, *Ah|SLD2.3*, *Ah|SLD2.4*, *Ah|SLD2.5*, and *Ah|SLD2.6* respond to drought stress. Taken together, these results provide significant insight into the potential functional roles of the *FAD* genes. A comprehensive analysis will further help screen *FAD* candidate genes for functional identification and provide resources and references for improving the agronomic traits and drought resistance of peanuts.

## Figures and Tables

**Table 1:** Conserved motifs and conserved motif sets of ω-3 desaturases, ω-6 desaturases, ω desaturases, ADSs (first/Arabidopsis desaturases), DSDs (sphingolipid/dihydroceramide desaturases) and SLDs (front-end desaturases/sphingoid long-chain bases at Δ8 desaturases), respectively (shaded part).

Subfamily	Gene	Number of Genes	Conserved Motifs																			Number of Motifs
ω-3 desaturase	FAD3	4				7		11	3	18	2	17	9	22	35	12	6	25	1	16		14
	FAD3	2			31	7		8	3	18	2	17	9	22	35	12	6	25	1	16		15
	FAD3	2		1	31	7		8	3	18	2	17	9	22	35	12	6	25	1	16		16
	FAD8	4	26	8	30	7		11	3	18	2	17	9	22	35	12	6	25	1	16		17
	FAD7	4	26	28	19	7		11	3	18	2	17	9	22	35	12	6	25	1	16		17
Motif set 1, shared in ω-3 desaturases						7			3	18	2	17	9	22	35	12	6	25	1	16		
ω-6 desaturase	FAD2	4				7	34	11	3	18	2	23	9	27		12	6	25	1	20		14
	FAD2	2			30	7	34	11	3	18	2	23	9	27		12	6	25	1	20		15
	FAD2	2			8	7	34	11	3	18	2	23	9	27		12	6	25	1	20		15
	FAD2	2				7	34	11	3	18	2	23	9	27		12	6	25	1	20		14
	FAD2	2				7	34	11	3	18	2	23	9	27		12	6	25	1			13
	FAD6	4				7	33		3		2	32	13			12	18	25	1	17	35	12
Motif set 2, shared in ω-6 desaturases						7	34	11	3	18	2	17	9	27		12	6	25	1	20		
Motif set 3, shared in ω desaturases						7			3	18	2		9			12	6	25	1			
First desaturase	ADS	2	31	2	19	30	8	21	28	3	32	33	24	27								12
	ADS	4	31	2	20	30	8	21	28	3	32	33	24	27								12
Motif set 4, shared in ADSs						30	8	21	28	3	32	33	24	27								
Sphingolipid desaturase	DSD	4			20	30	32	25	3	20	16	29	1	31								10
Frond-end desaturase	SLD	19	19	5	13	15	3	21	2	11	8	14	4	18	25	1	10	22				16
	SLD	1			13	15	3	21	2	11	8	14	4	18	25	1	10	22				14
Motif set 5, shared in SLDs					13	15	3	21	2	11	8	14	4	18	25	1	10	22				

## Data Availability

The genome and annotation gff3 files of *A. duranensis* (*Ad*, GCF_000817695.2), *A. ipaensis* (*Ai*, GCF_000816755.2), and *A. hypogaea* (*Ah*, GCA_003086295.2) were downloaded from the NCBI database (National Center for Biotechnology Information. Available online: https://www.ncbi.nlm.nih.gov/assembly/ (accessed on 12 July 2020)). The published FAD protein sequences of *Arabidopsis thaliana* and *Oryza sativa* were obtained from the TAIR (release 10, The Arabidopsis Information Resource (TAIR). Available online: http://www.arabidopsis.org/index.jsp (accessed on 22 July 2020)) and RGAP (release 7, Rice Genome Annotation Project (RGAP). Available online: http://rice.plantbiology.msu.edu/index.html (accessed on 22 July 2020)) databases, respectively. The SRA data of NH5 (drought-tolerant) and FH18 (drought-sensitive) transcriptomes were downloaded from the NCBI database (SRA accession: PRJNA657965). The experimental research on plants in this study complies with institutional, national, or international guidelines and the Convention on the Trade in Endangered Species of Wild Fauna and Flora.

## Supplementary Materials

The following supporting information can be downloaded at: https://www.mdpi.com/article/10.3390/genes13101718/s1, Table S1: Specific primers for qRT-PCR; Table S2: List of fatty acid desaturase genes identified in *Arachis* and their sequence properties; Table S3: Information of motifs in fatty acid desaturase genes; Table S4:Colliner genepairs of fatty acid desaturase among three peanut species; Table S5: Duplicated fatty acid desaturase genepairs in each of three Arachis species; Table S6: The Ka/Ks ratios of *FAD* pairs between diploid and tetraploid peanuts; Table S7: Details for predicted miRNA targeting *Ah|FADs*.
